# An Arabic gamified quiz to improve parental recognition of early developmental red flags: a pre-post pilot study

**DOI:** 10.3389/fped.2026.1858892

**Published:** 2026-06-10

**Authors:** Nisreen Al Awaji, Alanoud Alhasawi, Yara Zakri, Layan Al Dayel, Lama Alhamlan, Rawabi Almutairi

**Affiliations:** Department of Health Communication Sciences, College of Health and Rehabilitation Sciences, Princess Nourah bint Abdulrahman University, Riyadh, Saudi Arabia

**Keywords:** Arabic, developmental red flags, early identification, early intervention, feeding difficulties, gamification, parental awareness, speech and language development

## Abstract

**Introduction:**

Early identification of speech, language, hearing, social communication, and feeding difficulties depends substantially on parental recognition of developmental red flags. In Saudi Arabia, limited parental awareness may contribute to delayed referral and intervention. This pilot study evaluated the preliminary effectiveness of a 25-item Arabic gamified quiz in improving parental recognition of early developmental red flags in children from birth to 3 years of age.

**Methods:**

A single-session pre-post interventional design was used. Parents completed a pre-test, engaged with the Arabic gamified quiz, and then completed a post-test during the same online session. The quiz was designed as a multiple-choice, scenario-based educational tool with immediate explanatory feedback. Seventy-nine participants completed both assessments and were included in the matched analysis.

**Results:**

Mean knowledge scores increased significantly from 78.6% to 92.5% (*p* < 0.001), with a large effect size (Cohen's d = 1.04). Most participants (87.3%) demonstrated improved post-test scores.

**Discussion:**

These findings suggest that a brief Arabic gamified quiz may be a promising caregiver-facing educational tool for improving recognition of early developmental red flags in Arabic-speaking populations. Further research is needed to assess long-term knowledge retention, behavioral impact, and broader implementation.

## 1 Introduction

The early identification of speech, language, and feeding difficulties during the initial years of life is of paramount importance, as timely intervention can substantially improve developmental outcomes and mitigate the long-term consequences of late diagnosis on learning, social participation, and family well-being (1–3). In this context, red flags are defined as early warning signs in speech, language, and feeding development that may signal underlying communication or swallowing disorders if not addressed (4). Given that many of these early signs manifest in daily routines, such as vocal play, responsiveness to sounds, and mealtime behaviors, parents and caregivers are uniquely positioned to be the first to notice potential concerns (5) consequently, parental awareness represents a crucial leverage point for facilitating earlier detection and referral for specialized services (6).

Research has consistently shown that parental education and structured developmental guidance can enhance the recognition of early developmental concerns and increase the likelihood of seeking timely intervention services (7–10). A comprehensive global systematic review and meta-analysis of parenting interventions in the first three years of life underscored the importance of empowering caregivers with developmentally appropriate knowledge and skills to support early child development (9). Meta-analyses examining parent-implemented language interventions (8, 10) and family-centered help-giving practices (7, 10) have similarly demonstrated the effectiveness of parental education across diverse populations and intervention contexts. Research on family-centered early childhood intervention programs further supports the positive impact of parental involvement on both parent and child outcomes (11). However, despite the growing international focus on caregiver-facing educational resources and recent expansion of digital health initiatives in Saudi Arabia and the broader Middle East region (12), there remains a notable scarcity of culturally and linguistically tailored digital and gamified materials specifically designed for parental education about early childhood developmental red flags in Arabic-speaking contexts. While gamified learning platforms have been successfully implemented for Arabic language education in the region (13), similar tools targeting parental awareness of developmental red flags are limited. In these settings, awareness patterns may be influenced by local language nuances, health literacy levels, and the specific pathways to accessing healthcare services.

Within Saudi Arabia, a growing body of evidence indicates that gaps in parental knowledge are prevalent and may contribute to delays in both the recognition of developmental issues and the subsequent referral for assessment and therapy. For instance, a study conducted in the Eastern Region of the country revealed that parents had limited awareness of speech and language milestones and red flags, with many expressing uncertainty about what constitutes typical development (14). Similarly, research from primary healthcare centers in Riyadh reported that a substantial proportion of children with language impairments were not identified by their parents, highlighting the limitations of relying solely on informal observation without the aid of structured guidance (15). Further studies have reinforced the broader challenge of limited parental knowledge regarding developmental milestones and early identification (16), and various factors associated with parental knowledge in Saudi settings have been described (15). Collectively, these findings point to a persistent gap between parents’ informal observations and their understanding of typical developmental trajectories, which can impede timely action.

Traditional awareness campaigns, which often depend on the passive delivery of information, may not be sufficient to sustain attention or promote effective knowledge retention. In contrast, gamification, the application of interactive elements such as feedback, challenges, and progress indicators, has emerged as a promising approach to increase engagement and reinforce learning. Gamified learning designs can align effectively with principles of adult learning and social interaction by presenting educational content in problem-centered, relevant scenarios and by supporting the learning process through immediate feedback (17, 18). This approach is particularly timely given the rapid expansion of Arabic digital platforms and the increasing emphasis on digital innovation and preventive health awareness at a national level.

In light of the limited availability of culturally adapted Arabic gamified tools designed to enhance parental recognition of early speech, language, and feeding red flags, this study was developed to create and evaluate an Arabic gamified quiz for parents of children aged from birth to 3 years. Utilizing a pre-post study design, we aimed to determine whether parents’ recognition of these red flags improved following their engagement with the gamified tool and to explore which developmental domains exhibited the most significant gains in knowledge. The findings from this pilot study are intended to inform the development of scalable, caregiver-facing educational tools and to support broader early identification initiatives in Saudi Arabia and other Arabic-speaking regions.

## 2 Materials and methods

### 2.1 Study design

This study employed a single-session pre–post pilot interventional design to evaluate the preliminary effectiveness of a 25-item Arabic, parent-facing gamified quiz in improving parental recognition of early speech, language, hearing, social communication, and feeding red flags in children from birth to 3 years of age. A repeated-measures approach was used, whereby each participant completed the same 25-item knowledge assessment before and after exposure to the intervention during the same online session. The study was designed as a pilot study intended to assess the feasibility and preliminary educational impact of the intervention rather than long-term behavioral change or child developmental outcomes.

### 2.2 Participants and recruitment

The target population comprised Arabic-speaking parents or primary caregivers of children aged 0–3 years residing in Saudi Arabia. Participants were recruited using a non-probability convenience sampling strategy through online platforms and social media channels, including WhatsApp groups, X (formerly Twitter), and Instagram. Participation was voluntary, and no directly identifying information was collected. Responses were matched across the two time points using a participant identification code. A total of 149 individuals initiated the study, and 79 participants completed both the pre-test and post-test and were included in the matched pre–post analysis. The demographic and background variables collected included age, education, gender, number of children, age of the youngest child, prior awareness of the speech-language pathologist profession, previous child receipt of speech or feeding therapy, and the source through which participants learned about the questionnaire.

### 2.3 Development of the gamified quiz

#### 2.3.1 Tool design and content

A researcher-developed Arabic gamified quiz was created to assess and improve parental recognition of early developmental red flags. All participant-facing materials, including instructions, items, and feedback, were presented in Arabic. The instrument used a multiple-choice format and incorporated short, parent-oriented real-life scenarios. The study instrument was structured into two main parts. The first part collected demographic information. The second part contained the knowledge quiz and instructed participants to read each question carefully, select one answer only, and review the immediate feedback provided after responding. The instructions also stated that the responses were intended for educational and research purposes and that completion of the quiz required approximately 10–12 min.

The full Arabic research instrument contained 27 items across five domains: speech and language development (8 items), feeding and swallowing (6 items), hearing and responsiveness (5 items), social and cognitive communication (6 items), and professional awareness and referral pathways (2 items). For statistical analysis, only the 25 dichotomously scored knowledge items from the first four scored domains were included in the matched pre–post analysis. The complete 25-item Arabic gamified quiz, including all questions, answer options, correct answers, and explanatory feedback provided to participants, is provided in Supplementary Material 1 Appendix A.

#### 2.3.2 Theoretical framework

The intervention was informed by Knowles's Adult Learning Theory and Vygotsky's Social Interaction Theory. Adult Learning Theory guided the development of content that was problem-centered, relevant to parents’ everyday concerns, and appropriate for self-directed learning.

Vygotsky's Social Interaction Theory informed the use of structured explanatory feedback to support progressive learning throughout the quiz. These principles were operationalized through brief real-life scenarios, immediate feedback following each response, and sequential presentation of the 25 quiz items to reinforce parental recognition of developmental red flags.

#### 2.3.3 Gamification features

The intervention incorporated selected gamified and interactive learning elements rather than functioning as a fully game-based platform. Specifically, the 25-item Arabic quiz included immediate feedback after each response, concise explanations of the correct answers, scenario-based learning prompts, and a visually engaging interactive format. Each item was designed to reflect realistic situations that parents may encounter in daily life, allowing the quiz to function as both an assessment tool and a brief educational activity. In this way, the intervention promoted active user engagement while supporting parental learning about early developmental red flags.

### 2.4 Content validity and pilot testing

Content validity of the 25-item Arabic quiz was established through expert review by two certified Speech-Language Pathologists with experience in pediatric speech, language, and feeding disorders, one with more than 10 years of clinical experience and the other with 16 years of clinical experience. Each reviewer independently evaluated the quiz items for relevance, clarity, developmental appropriateness, and cultural suitability for Arabic-speaking parents.

Reviewer feedback was compiled by the research team, discussed, and used to refine the wording and structure of the 25-item quiz before finalization. Following expert review, the quiz underwent pilot refinement with a small group of parents to evaluate readability, usability, clarity of wording, and technical functionality. Feedback from this pilot phase informed minor revisions before final deployment.

### 2.5 Outcome measure and scoring

The primary outcome was parental knowledge of early speech, language, hearing, social communication, and feeding red flags. The outcome measure was based on a 25-item Arabic multiple-choice quiz. In the matched analytical dataset, each item was scored dichotomously as correct or incorrect. Total knowledge scores were calculated as the proportion of correctly answered items out of 25 and were expressed both as a proportion ranging from 0 to 1 and as a percentage ranging from 0% to 100%. In the dataset, this summary variable was recorded as “Average (%)”. Higher scores indicated greater parental recognition of developmental red flags.

### 2.6 Procedure

The study was delivered entirely online and completed in a single session. Participants accessed the study through an online link distributed via social media and completed all components independently. The study procedure consisted of three sequential stages: completion of the pre-test knowledge assessment, engagement with the 25-item Arabic gamified quiz, and completion of the post-test knowledge assessment. The quiz provided immediate explanatory feedback after each item, thereby functioning as both an assessment tool and a brief educational intervention. The estimated completion time of 10–12 min referred to the full study procedure, including the pre-test, quiz completion, and post-test.

### 2.7 Statistical analysis

All analyses were conducted at the participant level using paired statistical procedures to compare pre-test and post-test scores. Descriptive statistics were calculated for participant characteristics and knowledge scores.

Pre–post differences in total knowledge scores were assessed using a paired-samples t-test. To examine the robustness of the findings, a Wilcoxon signed-rank test was also performed as a non-parametric sensitivity analysis. The magnitude of the intervention effect was estimated using Cohen's d.

Exploratory subgroup analyses were conducted using separate paired-samples t-tests to examine whether score changes differed according to age group and education level. Because this was a pilot study, these subgroup analyses should be interpreted cautiously. Item-level analyses were also performed to assess changes in the proportion of correct responses across individual questions. All statistical tests were two-sided, and statistical significance was set at *p* < 0.05. All statistical analyses were conducted using IBM SPSS Statistics version 28.0.

### 2.8 Ethical considerations

Ethical approval was obtained from the Institutional Review Board of Princess Nourah bint Abdulrahman University (IRB No. 25–0677). This study was conducted in strict accordance with the ethical principles for research involving human participants. Participation was voluntary and anonymous, and informed consent was obtained through an informed consent statement presented at the beginning of the online survey before participants could proceed with the study. No personal data was collected, ensuring the privacy and confidentiality of all participants.

## 3 Results

### 3.1 Participant flow and demographic characteristics

A total of 149 participants initiated the study procedure, which consisted of completing a pre-test assessment, engaging with the Arabic gamified quiz, and completing a post-test during the same online session. Of these, 79 participants completed all study components and were included in the matched pre–post analysis.

Each participant contributed two observations (pre-test and post-test), yielding a total of 158 survey observations across both time points. All analyses were conducted at the participant level using paired statistical procedures. For clarity, the term sample refers to the number of unique participants included in the analysis (*n* = 79).

The demographic characteristics of the analyzed sample are presented in Table 1. The sample was predominantly female (91.1%). Approximately half of the sample (49.4%, *n* = 39) were aged 25 and 34 years, with an additional 32.9% (*n* = 26) aged 35-44 years. The majority of participants held a bachelor's degree (65.8%).

**Table 1:** Demographic characteristics of participants (*n* = 79).

Demographic Variable	Category	n	%
Age Group	Less than 25 years	8	10.1
	25–34 years	39	49.4
	35–44 years	26	32.9
	45 and above	6	7.6
Education Level	Diploma degree	5	6.3
	High school graduate	13	16.5
	Bachelor degree	52	65.8
	Postgraduate studies	9	11.4
Gender	Female	72	91.1
	Male	7	8.9

n, number of participants; %, percentage.

To examine potential attrition bias, the demographic characteristics of participants who completed both assessments were compared with those who did not complete the study. Of the 149 participants who initiated the study, 79 (53.0%) completed both the pre-test and post-test, while 70 (47.0%) did not complete both assessments. Demographic comparison between completers and dropouts revealed several notable differences: female participants comprised 91.1% of completers compared to 65.7% of dropouts; participants aged 25–34 years had the highest completion rate (65.0%), while younger participants (< 25 years) had the lowest (32.0%); and 65.8% of completers held a bachelor's degree compared to 50.0% of dropouts. These demographic differences are presented in Table 2.

**Table 2:** Demographic comparison.

Demographic Characteristic	Completers (*n* = 79)	Dropouts (*n* = 70)	Total (*n* = 149)
Gender			
Female	72 (91.1%)	46 (65.7%)	118 (79.2%)
Male	7 (8.9%)	23 (32.9%)	30 (20.1%)
Age Group			
< 25 years	8 (10.1%)	17 (24.3%)	25 (16.8%)
25–34 years	39 (49.4%)	21 (30.0%)	60 (40.3%)
35–44 years	26 (32.9%)	22 (31.4%)	48 (32.2%)
45 + years	5 (6.3%)	10 (14.3%)	15 (10.1%)
Education Level			
Diploma	5 (6.3%)	4 (5.7%)	9 (6.0%)
Secondary	13 (16.5%)	22 (31.4%)	35 (23.5%)
Bachelor's	52 (65.8%)	35 (50.0%)	87 (58.4%)
Graduate	9 (11.4%)	10 (14.3%)	19 (12.8%)

n, number of participants; %,  percentage. Dropout demographics are estimated based on proportional scaling from the participant database.

### 3.2 Overall knowledge improvement

The primary outcome of this study was the change in parental knowledge of early speech, language, and feeding red flags from pre-test to post-test. The descriptive statistics for this outcome are summarized in Table 3.

**Table 3:** Descriptive statistics for pre-test and post-test scores (*n* = 79).

Measure	Pre-test	Post-test	Difference
Mean (SD)	0.786 (0.161)	0.925 (0.131)	0.139 (0.134)
Mean % (SD)	78.6% (16.1%)	92.5% (13.1%)	13.9% (13.4%)
Median	80%	92%	12%
Range	0.24–1.00	0.24–1.00	–0.12 to +0.60

**Figure 1:**
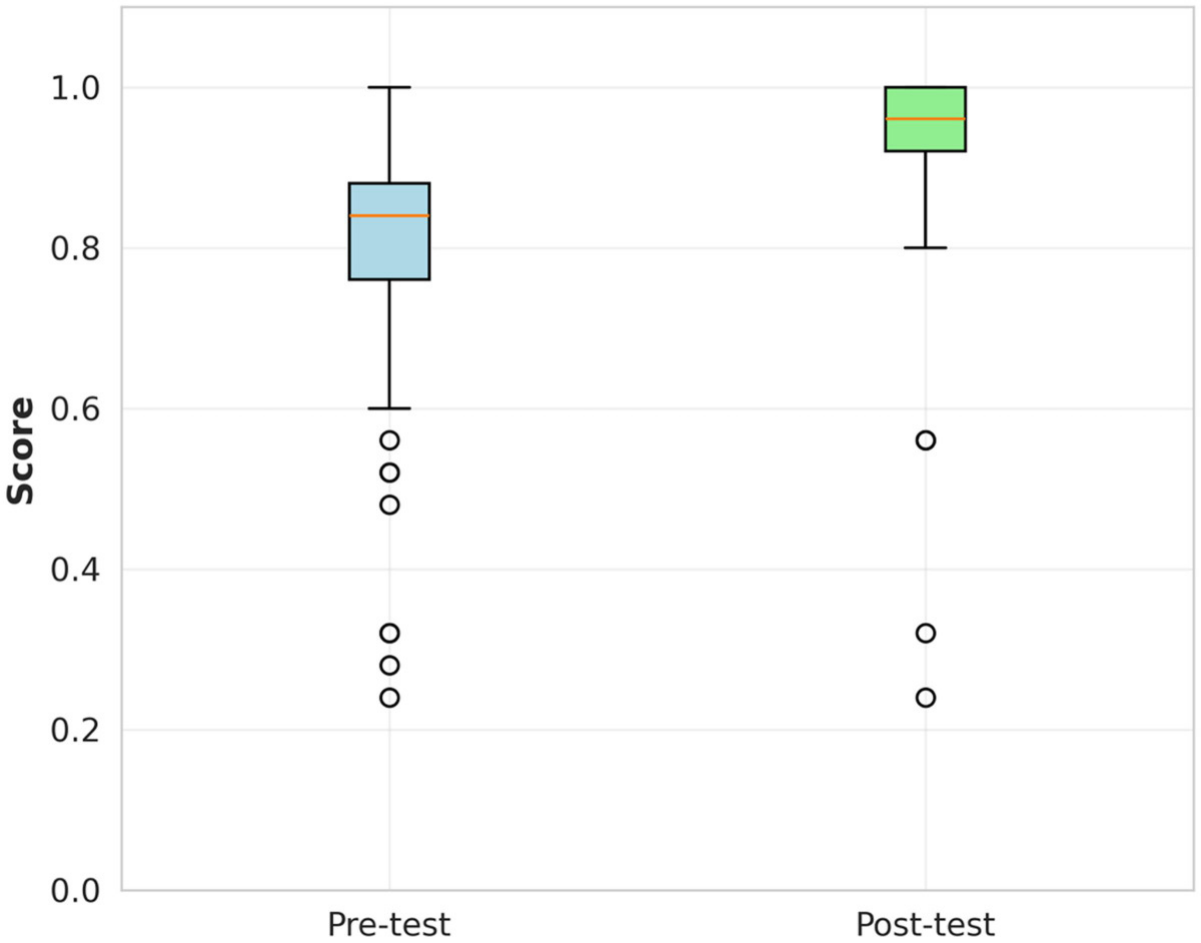
Comparison of pre-test (M = 78.6%, SD = 16.1%) and post-test (M = 92.5%, SD = 13.1%) scores (*n* = 79). Mean improvement = 13.9 percentage points.

The mean scores increased from 78.6% (SD = 16.1) at pre-test to 92.5% (SD = 13.1) at post-test, which corresponds to a mean improvement of 13.9 percentage points (SD = 13.4). The median scores also increased from 80% to 92%. The individual score changes ranged from a decrease of 12.0 percentage points to an increase of 60.0 percentage points. The distribution of the pre-test and post-test scores is illustrated in Figure 1.

### 3.3 Statistical significance and effect size

A paired-samples t-test revealed a statistically significant increase in knowledge following the intervention, with t(78) = 9.23 and *p* < 0.001. A Wilcoxon signed-rank test, which was conducted as a non-parametric sensitivity analysis, yielded comparable results (T-negative = 104.5, *p* < 0.001). The magnitude of the effect was determined to be large, with a Cohen's d of 1.04.

**Figure 2:**
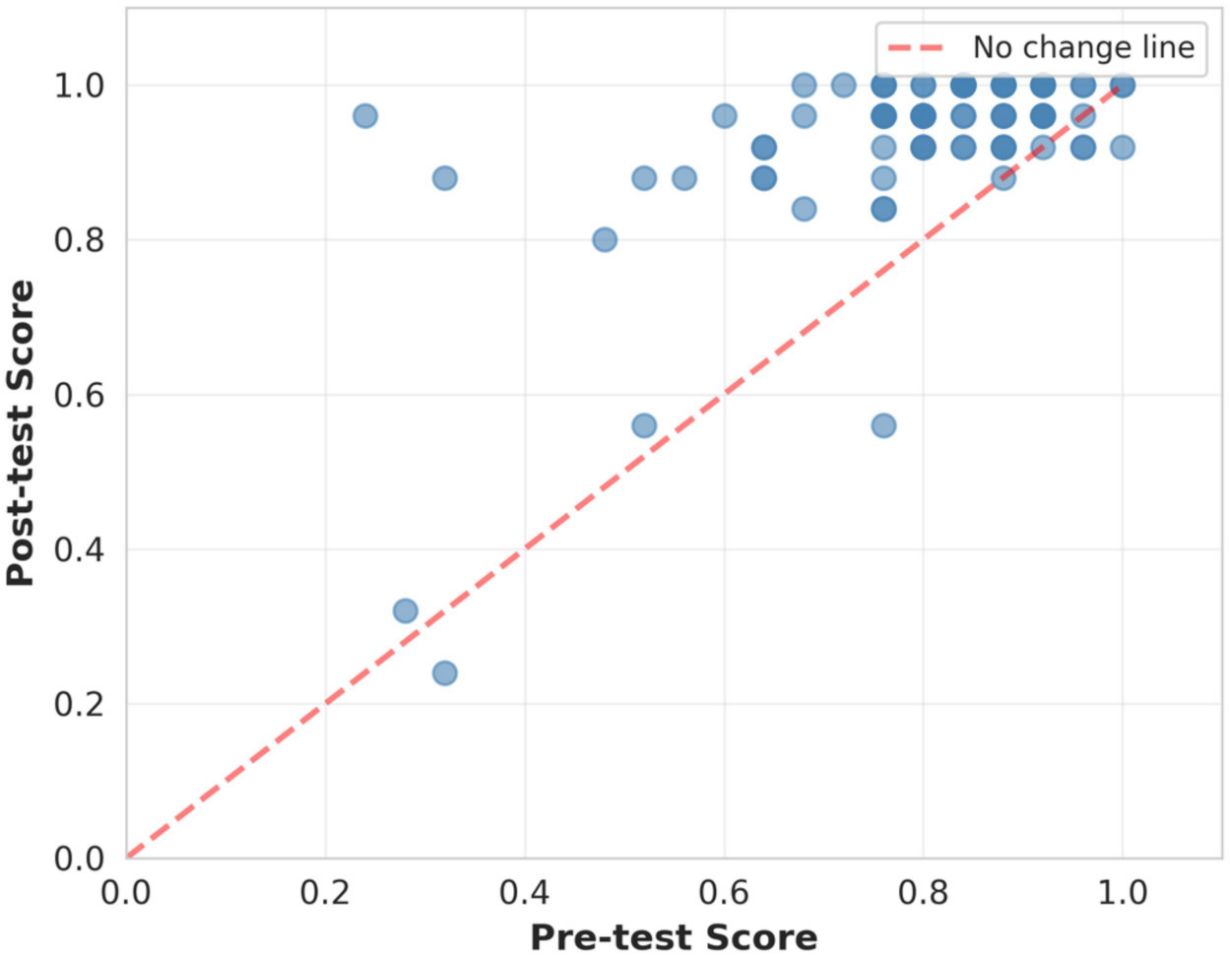
Individual participant trajectories showing pre-test vs. post-test performance (*n* = 79). The red dashed line indicates no change. Points above the line indicate improvement.

The individual participant trajectories from pre-test to post-test are shown in Figure 2. The majority of the data points lie above the line of no change, which provides a visual representation of the score improvement.

### 3.4 Distribution of participant-level changes

Participants were categorized based on the change in their scores between the pre-test and post-test, being classified as showing improvement, no change, or a decline. Of the 79 participants, 69 (87.3%) demonstrated improved scores, 5 (6.3%) showed no change in their scores, and 5 (6.3%) showed a decrease.

To examine the participants who showed no change or decline (*n* = 10, 12.7%), we analyzed their baseline (pre-test) scores. The mean pre-test score for this non-improving subgroup was 87.60% (SD = 20.87%), with a range of 32% to 100%. As shown in Table 4, this analysis revealed that a ceiling effect explains the lack of improvement for the majority of this group: 7 participants (70%) had high baseline scores (≥90%), including three at the maximum (100%). However, ceiling effect does not adequately explain the lack of improvement for all non-improving participants. Three participants (30%) had baseline scores below 90%, including two who showed declines despite having room for improvement (76%→56% and 32%→24%).

**Table 4:** Baseline score categories of non-improving participants (*n* = 10).

Baseline Score Category	n	%	Mean Pre-Test	Mean Change
High (≥90%)	7	70	97.14%	−2.86%
Moderate (70–89%)	2	20	82.00%	−10.00%
Low (<70%)	1	10	32.00%	−8.00%
Total	**10**	**100**	**87**.**60%**	**−3**.**20%**

Ceiling effect explains lack of improvement for high baseline group (70%). Alternative explanations needed for moderate and low baseline groups (30%).

**Figure 3:**
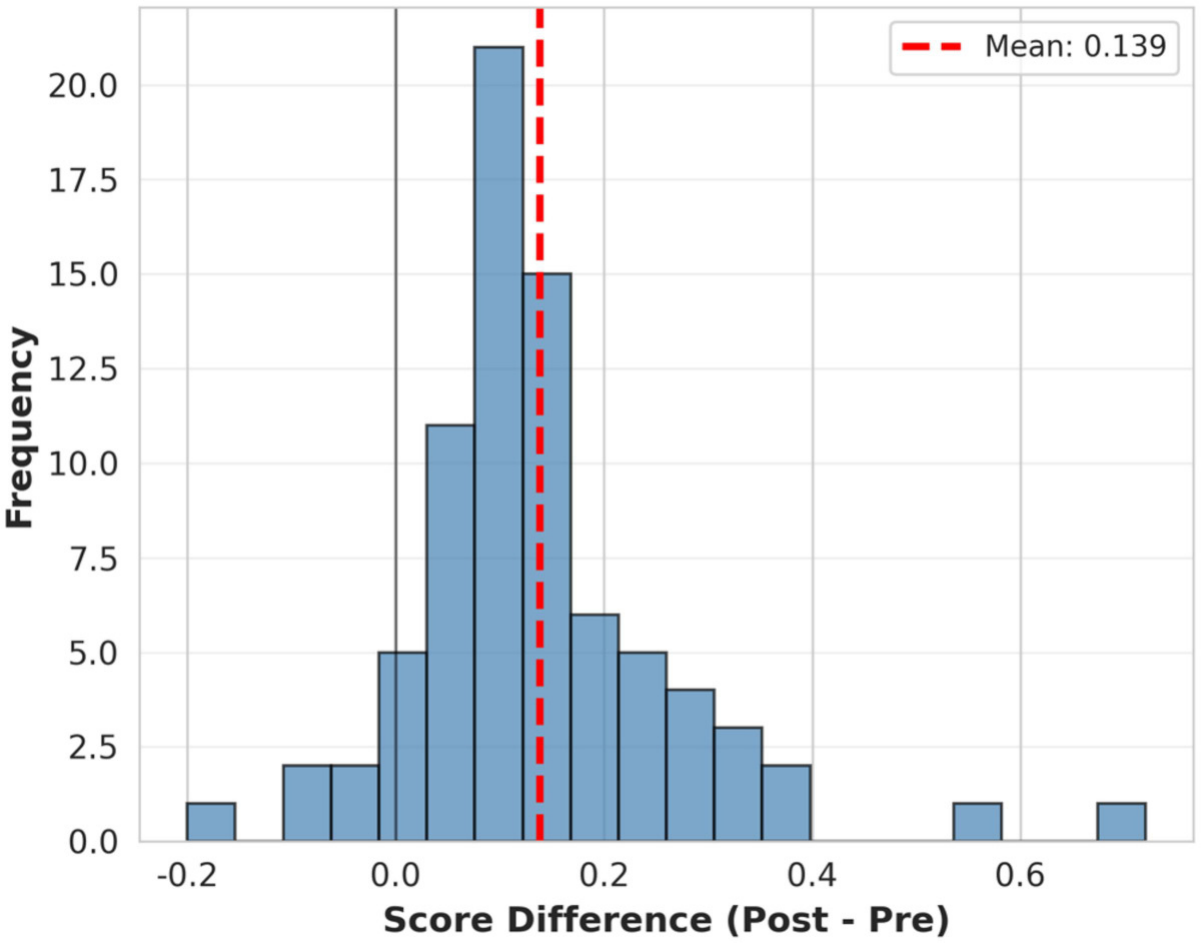
Distribution of score changes from pre-test to post-test (*n* = 79). Mean change = 13.9 percentage points (SD = 13.4%), median = 12.0 percentage points.

**Figure 4:**
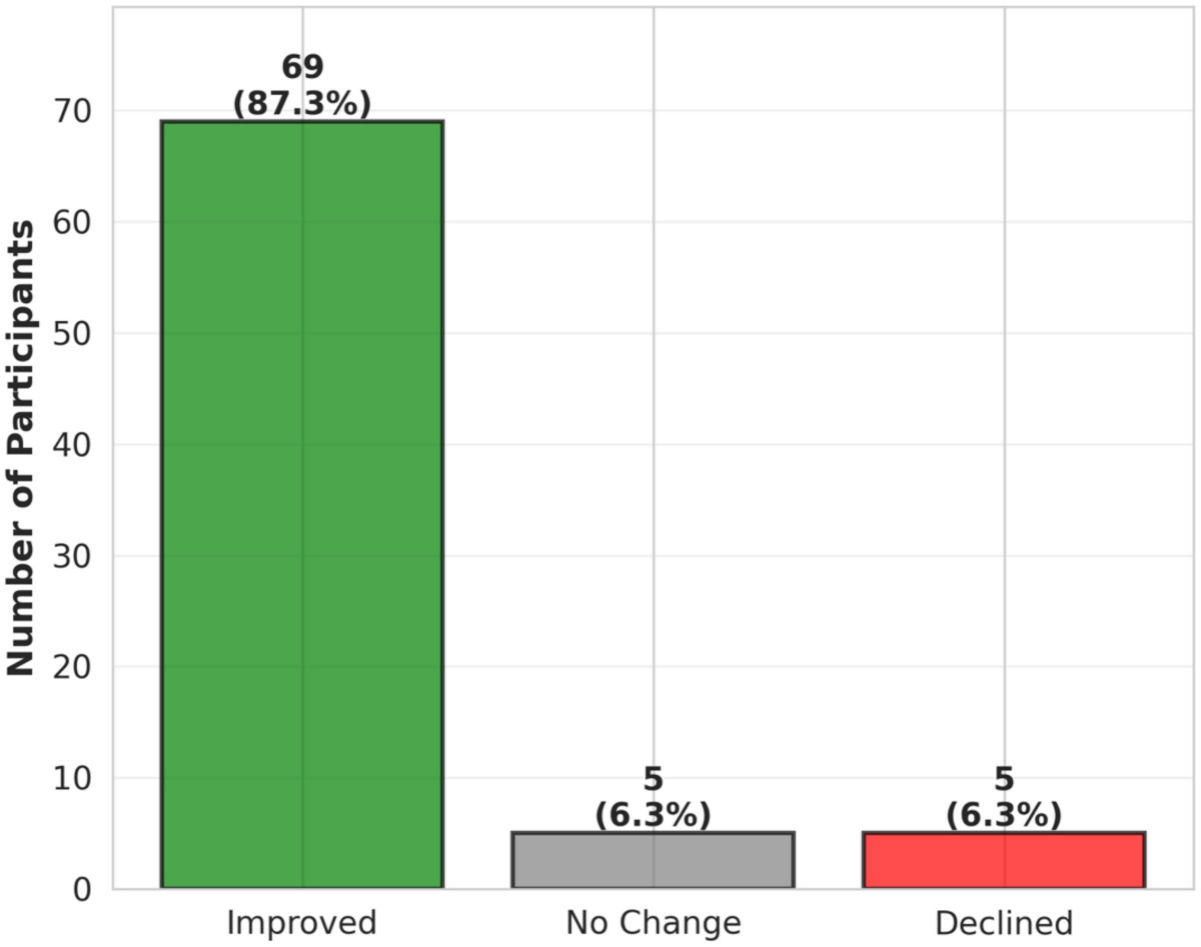
Participant improvement distribution (*n* = 79): 69 improved (87.3%), 5 no change (6.3%), 5 declined (6.3%).

The distribution of the score changes is presented in Figure 3, and the categorical distribution of the participant outcomes is illustrated in Figure 4.

### 3.5 Demographic subgroup analyses

To explore whether the knowledge gains differed across various demographic characteristics, separate paired-samples t-tests were conducted for different age groups and education levels.

#### 3.5.1 Age groups

Statistically significant pre-post improvements were observed across all age groups, as detailed in Table 5. The mean improvements ranged from 12.3 to 17.0 percentage points. Participants aged less than 25 years showed a mean increase of 17.0% (*p* = 0.0071), those aged 25–34 years improved by 14.3% (*p* < 0.001), participants aged 35–44 years improved by 12.3% (*p* = 0.001), and participants aged 45 years and above showed an improvement of 16.0% (*p* = 0.0309). Notably, the youngest and oldest age groups demonstrated the largest improvements, while middle-aged participants showed slightly smaller gains.

**Table 5:** Paired t-test results by age group (*n* = 79).

Age Group	n	Pre-test M (%)	Post-test M (%)	Improvement (%)	t-statistic	*p*-value
Less than 25 years	8	77.5	94.5	+17.0	3.76	0.0071
25–34 years	39	80.6	94.9	+14.3	6.37	<0.001
35–44 years	26	79.9	92.2	+12.3	4.62	0.001
45 and above	5	68.8	84.8	+16.0	3.13	0.0309

Statistical significance was set at *p* < 0.05.

#### 3.5.2 Education level

Improvements were observed across all education levels, as shown in Table 6. Statistically significant gains were found among participants with bachelor's degrees (mean increase = 15.3 percentage points, *p* < 0.001) and diploma degrees (mean increase = 14.4 percentage points, *p* = 0.0250). Participants with a high school education (mean increase = 10.9%, *p* = 0.0991) and those with postgraduate education (mean increase = 8.0%, *p* = 0.0618) did not show statistically significant changes in their scores. The lack of statistical significance in the postgraduate group may reflect a ceiling effect, as this group had the highest baseline mean score (84.0%), leaving limited room for improvement.

**Table 6:** Paired t-test results by education level (*n* = 79).

Education Level	n	Pre-test M (%)	Post-test M (%)	Improvement (%)	*p*-value
High school graduate	13	73.1	84.0	+10.9	0.0991
Diploma degree	5	65.6	80.0	+14.4	0.0250
Bachelor degree	52	79.3	94.6	+15.3	<0.001
Postgraduate studies	9	84.0	92.0	+8.0	0.0618

Statistical significance was set at *p* < 0.05.

#### 3.5.3 Item-Level analysis

Changes in the correct response rates were examined for each of the individual quiz items. Improvements were observed in 24 of the 25 questions. The mean improvement across all items was 13.9 percentage points, with the individual question-level improvements ranging from 0.0% to 57.0%.

**Figure 5:**
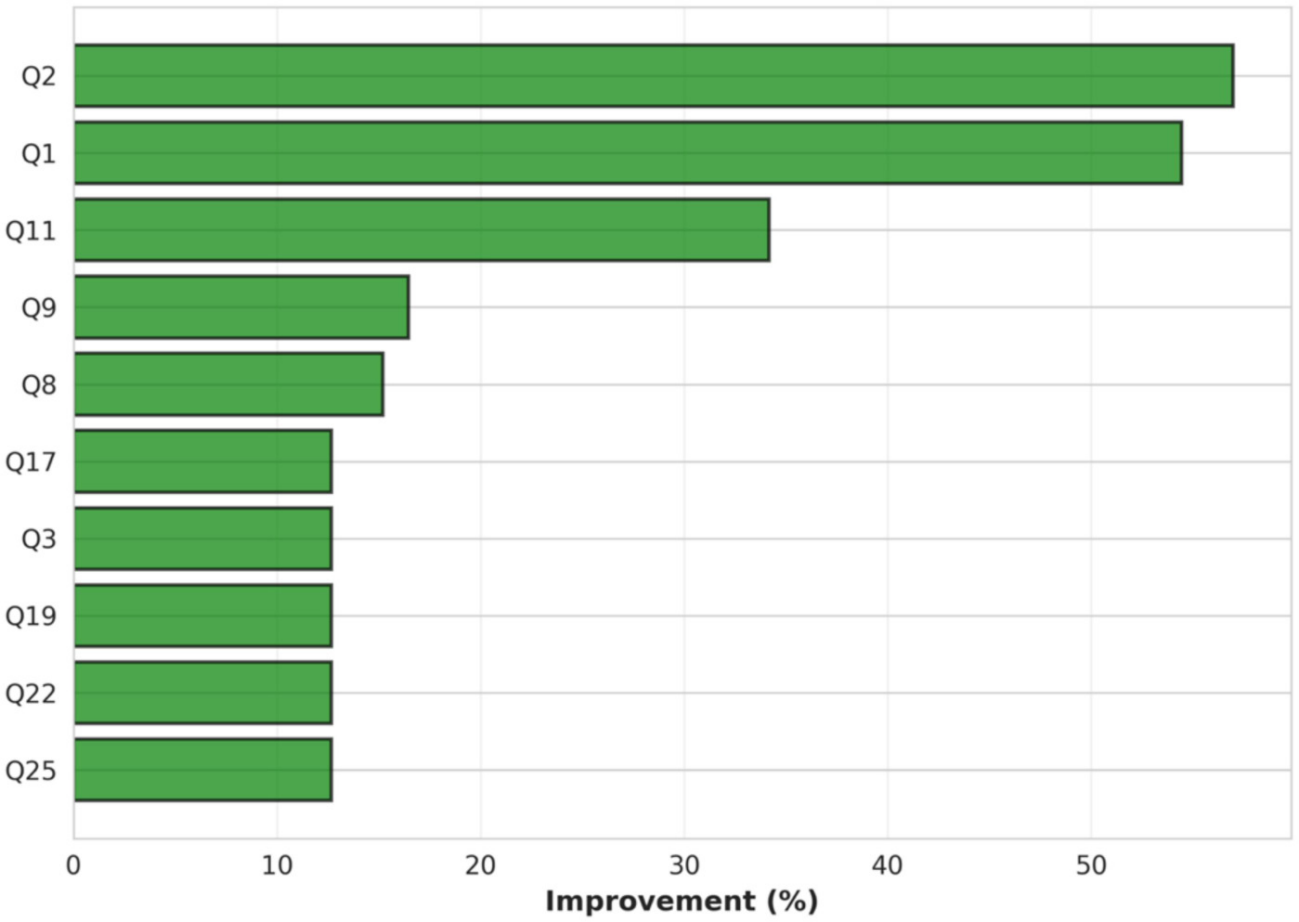
Top 10 questions ranked by improvement in correct response rate (*n* = 79). Q2 and Q1 showed the largest improvements (+57.0% and +54.4%, respectively).

**Figure 6:**
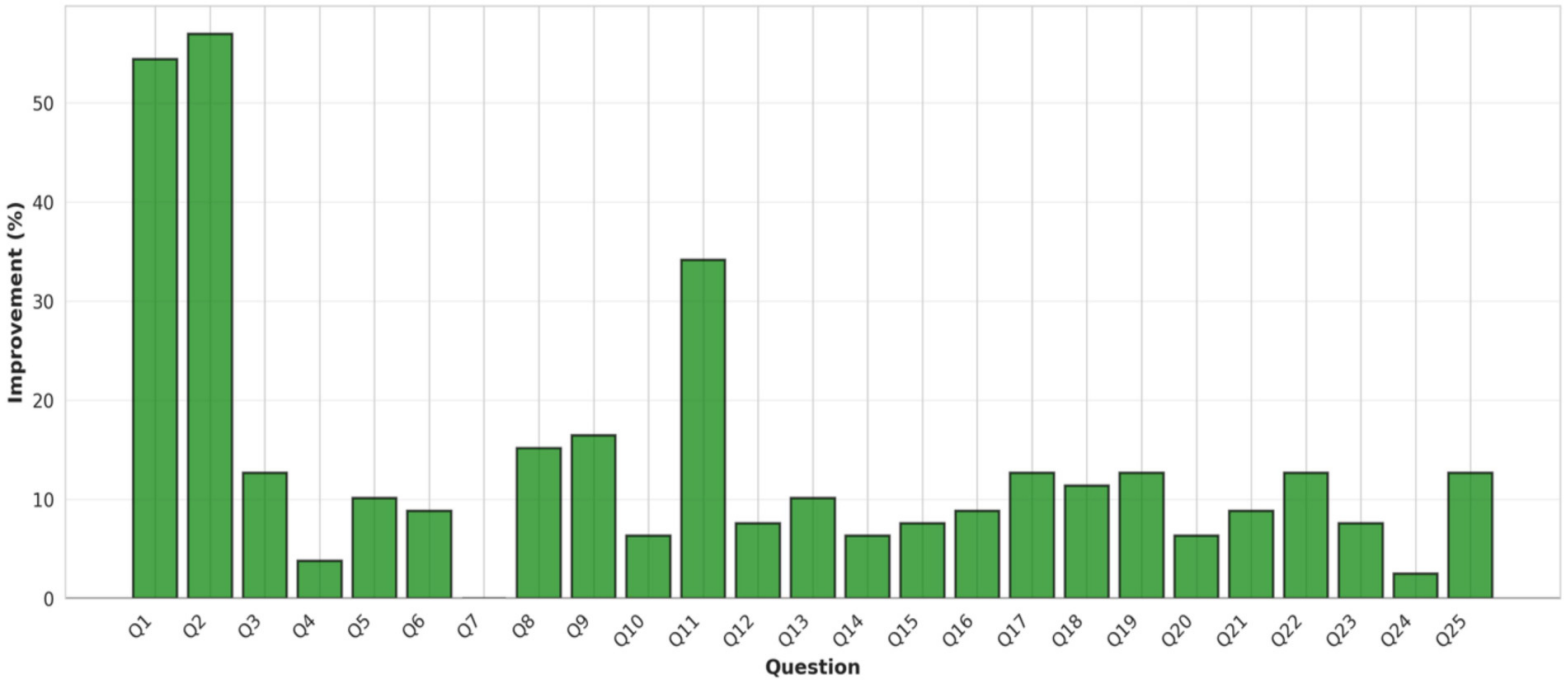
Improvement in correct response rate by question (*n* = 79). Green bars indicate positive improvement; 24 of 25 questions showed improvement.

The largest increases in correct responses were observed for questions related to early vocalizations (Question 2: +57.0%; Question 1: +54.4%; both *p* < 0.001), as depicted in Figure 5. The magnitude of the score changes across all questions is presented in Figure 6.

#### 3.5.4 Detailed item-level results

A complete summary of the correct response rates, percentage improvements, and statistical significance for all 25 questions is provided in Table 7. One item showed no change in the correct response rate, while all the remaining items demonstrated positive increases.

**Table 7:** Question-level analysis - correct response rates and improvement (*n* = 79).

Q	Topic	Pre-test (%)	Post-test (%)	Improvement (%)	*p*-value
Q1	Early vocalizations	34.2	88.6	+54.4	<0.001
Q2	Early vocalizations	30.4	87.3	+57.0	<0.001
Q3	Speech development	67.1	81.0	+13.9	0.0254
Q4	Speech development	81.0	84.8	+3.8	0.6725
Q5	Speech development	75.9	91.1	+15.2	0.0254
Q6	Speech development	62.0	75.9	+13.9	0.1378
Q7	Speech development	93.7	93.7	0.0	1.000
Q8	Speech comprehension	49.4	64.6	+15.2	0.0772
Q9	Feeding	79.7	96.2	+16.5	0.0033
Q10	Feeding	88.9	95.1	+6.2	0.2421
Q11	Feeding	50.6	84.8	+34.2	<0.001
Q12	Feeding	89.9	96.2	+6.3	0.2112
Q13	Feeding	81.0	91.1	+10.1	0.1378
Q14	Feeding	89.9	96.2	+6.3	0.2112
Q15	Hearing	91.1	97.5	+6.3	0.1697
Q16	Hearing	91.1	97.5	+6.3	0.1697
Q17	Hearing	91.1	97.5	+6.3	0.1697
Q18	Hearing	91.1	97.5	+6.3	0.1697
Q19	Social interaction	70.9	84.8	+13.9	0.0254
Q20	Social interaction	91.1	97.5	+6.3	0.1697
Q21	Social interaction	64.6	78.5	+13.9	0.0772
Q22	Social interaction	81.0	91.1	+10.1	0.1378
Q23	Cognitive development	62.0	75.9	+13.9	0.1378
Q24	Professional awareness	94.9	97.5	+2.5	0.6772
Q25	Professional awareness	96.2	98.7	+2.5	0.6772

p-values were obtained using paired-samples tests; statistical significance was set at *p* < 0.05

The most substantial improvements were observed for questions related to early vocalizations (Q1-Q2). Items related to feeding (Q9-Q14) and social interaction (Q19-Q22) showed moderate improvements. In contrast, questions that addressed hearing and professional awareness (Q15-Q18, Q24-Q25) demonstrated smaller changes, which were largely a reflection of the high baseline accuracy for these items. This pattern is consistent with a ceiling effect, where participants already demonstrated strong knowledge in these areas before the intervention.

## 4 Discussion

This pilot pre-post study demonstrated that a brief, Arabic gamified quiz significantly improved parental recognition of early speech, language, and feeding red flags in children aged from birth to 3 years. The magnitude, consistency, and pattern of these improvements align with and extend the existing literature on early identification, parental health literacy, and the use of digital health education.

### 4.1 Overall knowledge gains in relation to prior evidence

The observed mean improvement of 13.9 percentage points, coupled with a large effect size (Cohen's d = 1.04), is consistent with previous studies that have shown that targeted educational interventions can meaningfully enhance parental knowledge of early developmental markers (19, 20). Parental awareness is a critical determinant of early referral, as parents are often the first to notice developmental concerns, particularly in the domains of communication and feeding (21).

Importantly, the magnitude of the improvement observed in the present study is comparable to, and in some cases exceeds, that which has been reported in traditional parent education programs. These programs are typically more resource-intensive and are often delivered over multiple sessions (22). This finding lends support to the growing body of evidence suggesting that brief, digitally delivered interventions can produce learning gains that are similar to those of more time-intensive formats, particularly when the content is focused and contextually relevant (23, 24).

### 4.2 Gamification and digital learning effects

The strong post-intervention gains that were observed in this study are in alignment with the literature that indicates that gamified learning can enhance engagement, motivation, and short-term knowledge acquisition in health education contexts (25, 26). Gamification is particularly effective for lay audiences when complex clinical concepts, such as developmental milestones, are translated into simple and interactive decision points (27).

In the context of parental education, digital and mobile-based tools have been demonstrated to reduce barriers to access, increase the reach of educational materials, and support self-paced learning, especially among caregivers who may not otherwise be able to attend formal educational sessions (28). The findings of the present study extend this evidence to Arabic-speaking parents, a population that has remained underrepresented in the research on digital early development.

### 4.3 Demographic effects and health literacy considerations

Knowledge gains were observed across all age groups in this study, which is consistent with research suggesting that the benefits of digital health education are not limited to younger adults, provided that the user interface is intuitive and the content is clearly structured (29). This finding challenges the persistent assumption that older parents may be less responsive to digital interventions.

The differences that were observed across education levels in this study likely reflect baseline disparities in knowledge and the presence of ceiling effects, rather than a differential responsiveness to the intervention itself. Similar patterns have been reported in the health literacy literature, where participants with higher levels of education often demonstrate smaller absolute gains due to their higher pre-intervention scores (30). Conversely, participants with lower baseline knowledge may require repeated exposure or multimodal reinforcement to achieve comparable post-intervention gains, which is an important consideration for the future refinement of this tool. However, the subgroup analyses by age and education level should be interpreted with considerable caution given the limited statistical power in several subgroups. Specifically, the diploma-level (*n* = 5) and 45 + age group (*n* = 5) subgroups are particularly small, and results for these groups may be subject to chance variation. These exploratory findings are informative for hypothesis generation but should not be considered definitive. It should be noted that these demographic patterns may be partially influenced by the non-random attrition observed in the study, whereby younger and less-educated participants were more likely to drop out. This selection bias should be considered when interpreting subgroup differences. Future research with larger sample sizes would be needed to draw reliable conclusions about differential intervention effects across demographic subgroups and to consolidate or refine the subgroup categories as suggested by the current pilot data.

The analysis of non-improving participants provides important context for interpreting the overall findings. While a ceiling effect explains the lack of improvement for 70% of this group, alternative explanations such as participant fatigue, question ambiguity, or technical issues in the unmonitored online setting may contribute to the lack of improvement or decline observed in the remaining 30%. Future research should investigate these factors through post-intervention surveys or qualitative interviews.

#### 4.3.1 Ceiling effect and tool sensitivity

The post-test score distribution (Figure 1) reveals a compressed upper quartile, indicating a prominent ceiling effect for the overall tool. While this ceiling effect is consistent with the significant improvements observed in most participants, it also suggests potential limitations for the tool's sensitivity and discriminative ability, particularly when deployed among higher-educated or more knowledgeable populations.

Specifically, the ceiling effect may limit the tool's ability to differentiate between participants with varying levels of knowledge in higher-educated groups, where many individuals may already possess substantial baseline knowledge. In such populations, the tool may function more effectively as a screening or awareness-raising tool rather than as a comprehensive assessment.

These findings highlight the importance of considering population-specific characteristics when implementing the tool. Future research should: (1) evaluate the tool's performance in higher-educated populations, (2) explore tiered or adaptive versions that adjust difficulty based on baseline knowledge, (3) investigate alternative item formats to enhance sensitivity, and (4) determine the tool's most appropriate use cases across diverse populations

### 4.4 Item-level findings and developmental knowledge gaps

The largest improvements in knowledge were observed in the questions that were related to early vocalizations, which also had the lowest baseline accuracy. This finding is highly consistent with the prior literature, which indicates that parents often underestimate the clinical significance of pre-linguistic vocal behaviors, despite their strong predictive value for later speech and language outcomes (31, 32).

In contrast, the questions that were related to hearing and professional awareness showed smaller gains, which were largely due to the high baseline accuracy on these items. This mirrors the findings from population-level studies that have reported a relatively high level of parental awareness of newborn hearing screening and referral pathways, particularly in regions with established screening programs (33). However, the awareness of what constitutes an early communication red flag remains less developed, which underscores the importance of content prioritization in parental education initiatives.

The moderate improvements that were observed in the feeding-related items are also noteworthy. Feeding difficulties in infancy are frequently normalized or misattributed by caregivers, despite the strong associations that these difficulties have with neurodevelopmental and communication disorders (34). The gains that were observed in this area suggest that feeding-related red flags are amenable to brief educational interventions when they are explicitly addressed.

### 4.5 Implications for early identification and service pathways

From an early intervention perspective, the findings of this study reinforce the critical role of parent-mediated identification as a gateway to accessing timely services. Delays in recognition and referral remain a global challenge, particularly in the field of speech-language pathology, where “wait-and-see” approaches often persist despite the evidence that supports the benefits of early action (8).

In this context, scalable digital tools, such as the one evaluated in this study, may function as a first-line public health strategy that complements, rather than replaces, formal screening and developmental surveillance systems (35). The delivery of this tool in the Arabic language is particularly relevant, as the limited availability of culturally and linguistically adapted resources has been identified as a significant barrier to parental engagement in many non-English-speaking settings (36).

### 4.6 Limitations

The single-session pre-post design without a control group is a significant methodological limitation that precludes causal inference about the specific contribution of the gamified quiz. The observed knowledge gains may reflect test-retest practice effects, incidental learning from pre-test exposure, or other factors such as repeated exposure to content or the educational video, rather than the gamified component specifically. Additionally, the use of an immediate post-test assessment does not allow for conclusions about long-term knowledge retention or behavioral changes. These limitations are consistent with challenges commonly noted in pilot studies of digital health interventions and gamification research (25). To establish causal relationships and determine the specific mechanisms driving knowledge gains, future research should employ a randomized controlled trial design with distinct intervention and control groups, as well as a delayed post-test assessment to evaluate knowledge retention beyond the immediate post-intervention period.

The predominance of female and relatively well-educated participants in this study mirrors the patterns that are commonly seen in parental research more broadly, but it also limits the generalizability of the findings. While the use of convenience sampling through online platforms was employed, this approach is less likely to introduce digital literacy bias in the Saudi Arabian context, where internet penetration is exceptionally high at 99% among individuals aged 15–74 years (37, 38). However, the convenience sampling method may still introduce selection bias related to motivation to seek educational resources and parental engagement. Future research should deliberately target underrepresented caregiver groups and employ more diverse recruitment strategies in order to address the known inequities in health literacy and access to services.

The high mean pre-test score (78.6%) and near-ceiling baseline performance on hearing and professional awareness items (>90%) suggest that the recruited sample may not adequately represent caregivers with the greatest educational need. This pre-test ceiling effect likely attenuates the observed effect size and limits conclusions about the tool's utility in lower-knowledge populations, which are arguably the primary target of this intervention. The sample's relatively high baseline knowledge may have constrained the potential for improvement. Future research should deliberately recruit caregivers with lower baseline knowledge to determine whether larger effect sizes can be achieved in the target population.

The subgroup analyses by age and education level, while exploratory and informative, are limited by small sample sizes in several subgroups. Specifically, the diploma-level (*n* = 5) and 45 + age group (*n* = 6) subgroups are particularly small and have limited statistical power. Results for these subgroups should be interpreted cautiously and may not be reliable. Future research with larger sample sizes would be needed to draw definitive conclusions about differential intervention effects across demographic subgroups. Additionally, consolidating these small subgroups into broader categories (e.g., combining diploma and high school into ‘non-bachelor’ education, or combining 35–44 and 45 + into ‘35+’ age group) may improve statistical stability and provide more reliable estimates in future studies.

Attrition and Selection Bias: The study experienced a 47.0% dropout rate, with 70 of 149 participants who initiated the study not completing both assessments. While this dropout rate is not unusual for online interventions delivered through social media channels, it does warrant careful consideration of potential selection bias. Demographic analysis revealed that dropouts were more likely to be younger (24.3% < 25 years vs. 10.1% of completers), male (32.9% vs. 8.9%), and have high school education (31.4% vs. 16.5%). These differences suggest potential attrition bias, whereby the final sample of completers may not be fully representative of the initial population. Specifically, the findings may be less generalizable to younger and less-educated populations, as these groups had higher dropout rates. The non-random nature of attrition introduces selection bias that could affect the validity of our conclusions. Future studies should consider strategies to improve retention rates and should examine whether the observed knowledge gains are consistent across more diverse educational backgrounds and age groups.

## 5 Conclusions

The findings of this study align with and extend the existing evidence that brief, gamified digital interventions can meaningfully enhance parental knowledge of early developmental red flags. This study contributes novel evidence from an Arabic-speaking context and highlights the particular importance of early vocalizations and feeding behaviors as priority targets for parental education. When thoughtfully integrated into broader early identification frameworks, such tools have the potential to reduce delays in the recognition of developmental issues and support more timely access to essential intervention services. Future implementation efforts should prioritize accessibility and reach to underrepresented populations to maximize the public health impact of such tools. While subgroup analyses suggest potential differential effects by age and education level, these findings should be interpreted as exploratory given the limited sample sizes in some subgroups, and future research with larger samples is needed to confirm these patterns.

## Data Availability

The raw data supporting the conclusions of this article will be made available by the authors, without undue reservation.

## References

[1] Yoshinaga-Itano C. From screening to early identification and intervention: discovering predictors to successful outcomes for children with significant hearing loss. J Deaf Stud Deaf Educ. (2003) 8:11–30. 10.1093/deafed/8.1.11 15448044

[2] Yoshinaga-Itano C Sedey AL Coulter DK Mehl AL. Language of early- and later-identified children with hearing loss. Pediatrics. (1998) 102:1161–71. 10.1542/peds.102.5.1161 9794949

[3] Shriberg LD Gruber FA Kwiatkowski J. Developmental phonological disorders III. J Speech, Hear Res. (1994) 37:1151–77. 10.1044/jshr.3705.1151 7823558

[4] Glascoe FP. Parents’ concerns about Children's Development: prescreening technique or screening test? Pediatrics. (1997) 99:522–8. 10.1542/peds.99.4.522 9093291

[5] American Academy of Pediatrics. Identifying infants and young children with developmental disorders in the medical home: an algorithm for developmental surveillance and screening. Pediatrics. (2006) 118:405–20. 10.1542/peds.2006-1231 16818591

[6] Marshall J Coulter ML Gorski PA Ewing A. Parent recognition and responses to developmental concerns in young children. Infants Young Child. (2016) 29:102–15. 10.1097/IYC.0000000000000056

[7] Dunst CJ Trivette CM Hamby DW. Meta-analysis of family-centered help giving practices research. Ment Retard Dev Disabil Res Rev. (2007) 13:370–8. 10.1002/mrdd.20176 17979208

[8] Roberts MY Kaiser AP. The effectiveness of parent-implemented language interventions: a meta-analysis. Am J Speech Lang Pathol. (2011) 20:180–99. 10.1044/1058-0360(2011/10-0055) 21478280

[9] Jeong J Franchett EE Ramos de Oliveira CV Rehmani K Yousafzai AK. Parenting interventions to promote early child development in the first three years of life: a global systematic review and meta-analysis. PLoS Med. (2021) 18:e1003602. 10.1371/journal.pmed.1003602 33970913 PMC8109838

[10] Emmers D Jiang Q Xue H Zhang Y Zhang Y Zhao Y. Early childhood development and parental training interventions in rural China: a systematic review and meta-analysis. BMJ Glob Health. (2021) 6:e005578. 10.1136/bmjgh-2021-005578 PMC8381307 34417271

[11] Hughes-Scholes CH Gavidia-Payne S. Early childhood intervention program quality: examining family-centered practice, parental self-efficacy and child and family outcomes. Early Child Educ J. (2019) 47:719–29. 10.1007/s10643-019-00961-5

[12] Cheriyan G. How digital innovation is reshaping healthcare in the Middle East. World Economic Forum. (2024). Available online at: https://www.weforum.org/stories/2024/10/digital-innovation-reshaping-healthcare-middle-east/ (Accessed June 4, 2026).

[13] Almelhes SA. Gamification for teaching the arabic language to non-native speakers: a systematic literature review. Front Educ (Lausanne). (2024) 9:1371955. 10.3389/feduc.2024.1371955

[14] Alramadhan M Almatroushi N Mubarak A Alali J Tumayhi S Alarfaj A. Parental awareness, knowledge, and practices regarding speech and language delay in children from the eastern region of Saudi Arabia: a cross-sectional study. Cureus. (2025) 17:e81248. 10.7759/cureus.81248 40291292 PMC12030804

[15] Alakeely MH Alabbasi H Alohali L Aldughaither A. The ability of Saudi parents’ to detect early language delay in their children: a study in primary health care centers, king abdulaziz medical city, Riyadh, Saudi Arabia. Cureus. (2022) 14:e21448. 10.7759/cureus.21448 35223233 PMC8857898

[16] Alhwoaimel NA Almarzoug H Aldukhaini R Altamimi R Aldosre M Al-faris S. Parental knowledge of children's Motor development: a cross-sectional study in Saudi Arabia. Res Dev Disabil. (2023) 139:104552. 10.1016/j.ridd.2023.104552 37295126

[17] Lippitt GL Knowles MS Knowles MS. Andragogy in action: applying modern principles of adult learning. Jossey-Bass. (1984).

[18] Vygotsky LS. Mind in Society: The Development of Higher Psychological Processes. Cambridge, MA: Harvard University Press (1978).

[19] Glascoe FP. Evidence-based approach to developmental and behavioural surveillance using parents’ concerns. Child Care Health Dev. (2000) 26:137–49. 10.1046/j.1365-2214.2000.00173.x 10759753

[20] Zwaigenbaum L Bauman ML Choueiri R Kasari C Carter A Granpeesheh D. Early intervention for children with autism spectrum disorder under 3 years of age: recommendations for practice and research. Pediatrics. (2015) 136:S60–81. 10.1542/peds.2014-3667E 26430170 PMC9923898

[21] Ertem IO, World Health Organization. Developmental difficulties in early childhood: prevention, early identification, assessment and intervention in low- and middle-income countries: a review. World Health Organization. (2012). Available online at: https://iris.who.int/server/api/core/bitstreams/d013075d-528d-432c-beee-c73b3e0d88ce/content (Accessed June 4, 2026).

[22] Lipkin PH Macias MM Norwood KW Brei TJ Davidson LF Davis BE. Promoting optimal development: identifying infants and young children with developmental disorders through developmental surveillance and screening. Pediatrics. (2020) 145:e20193449. 10.1542/peds.2019-3449 31843861

[23] O'Connor S Hanlon P O'Donnell CA Garcia S Glanville J Mair FS. Understanding factors affecting patient and public engagement and recruitment to digital health interventions: a systematic review of qualitative studies. BMC Med Inform Decis Mak. (2016) 16:120. 10.1186/s12911-016-0359-3 27630020 PMC5024516

[24] Timpel P Oswald S Schwarz PEH Harst L. Mapping the evidence on the effectiveness of telemedicine interventions in diabetes, dyslipidemia, and hypertension: an Umbrella review of systematic reviews and meta-analyses. J Med Internet Res. (2020) 22:e16791. 10.2196/16791 32186516 PMC7113804

[25] Sardi L Idri A Fernández-Alemán JL. A systematic review of gamification in e-health. J Biomed Inform. (2017) 71:31–48. 10.1016/j.jbi.2017.05.011 28536062

[26] Johnson D Deterding S Kuhn KA Staneva A Stoyanov S Hides L. Gamification for health and wellbeing: a systematic review of the literature. Internet Interv. (2016) 6:89–106. 10.1016/j.invent.2016.10.002 30135818 PMC6096297

[27] Shorey S Ng YPM Danbjørg DB Dennis C Morelius E. Effectiveness of the ‘home-but not alone’ mobile health application educational programme on parental outcomes: a randomized controlled trial, study protocol. J Adv Nurs. (2017) 73:253–64. 10.1111/jan.13151 27650320

[28] Pretorius C Chambers D Coyle D. Young People's Online help-seeking and mental health difficulties: systematic narrative review. J Med Internet Res. (2019) 21:e13873. 10.2196/13873 31742562 PMC6891826

[29] Lesauskaitė V Damulevičienė G Knašienė J Kazanavičius E Liutkevičius A Janavičiūtė A. Older adults—potential users of technologies. Medicina (B Aires). (2019) 55:253. 10.3390/medicina55060253 PMC6631069 31181673

[30] Berkman ND Sheridan SL Donahue KE Halpern DJ Crotty K. Low health literacy and health outcomes: an updated systematic review. Ann Intern Med. (2011) 155:97–107. 10.7326/0003-4819-155-2-201107190-00005 21768583

[31] Oller DK Eilers RE Neal AR Schwartz HK. Precursors to speech in infancy. J Commun Disord. (1999) 32:223–45. 10.1016/S0021-9924(99)00013-1 10466095

[32] Paul D Roth FP. Guiding principles and clinical applications for speech-language pathology practice in early intervention. Lang Speech Hear Serv Sch. (2011) 42:320–30. 10.1044/0161-1461(2010/09-0079) 21060115

[33] Yoshinaga-Itano C. Early intervention after universal neonatal hearing screening: impact on outcomes. Ment Retard Dev Disabil Res Rev. (2003) 9:252–66. 10.1002/mrdd.10088 14648818

[34] Arvedson JC. Assessment of pediatric dysphagia and feeding disorders: clinical and instrumental approaches. Dev Disabil Res Rev. (2008) 14:118–27. 10.1002/ddrr.17 18646015

[35] World Health Organization. WHO guideline Recommendations on Digital Interventions for Health System Strengthening. Geneva: World Health Organization (2019). Available online at: https://www.who.int/publications/i/item/9789241550505 (Accessed June 4, 2026). 31162915

[36] DeWalt DA Hink A. Health literacy and child health outcomes: a systematic review of the literature. Pediatrics. (2009) 124:S265–74. 10.1542/peds.2009-1162B 19861480

[37] Communications and Information Technology Commission. Saudi Internet Report 2024. Communications and Information Technology Commission. (2024). Available online at: https://www.cst.gov.sa/en/media-center/news/N2025051200 (Accessed June 4, 2026).

[38] The General Authority for Statistics. Internet usage among individuals aged 15-74 years in Saudi Arabia. General Authority for Statistics. (2025). Available online at: https://www.stats.gov.sa/documents/20117/2435267/ICT+Access+and+Usage+2025-EN.pdf/d91fc7dd-2f2e-ee1a-248c-c8f38102ef01?t=176760367464538 (Accessed June 4, 2026).

