# Supplementary material for: An Arabic gamified quiz to improve parental recognition of early developmental red flags: a pre-post pilot study
Source: Front Pediatr. 2026 Jun 10;14:1858892. doi: 10.3389/fped.2026.1858892 (PMC13291250; doi:10.3389/fped.2026.1858892)
Supplement: Supplementary file 1 [file Supplementaryfile1.docx]

Appendix A: 25-Item Arabic Gamified Quiz on Early Speech, Language, and Feeding Red Flags

الملحق أ: استبيان مكون من 25 عنصراً حول علامات التحذير المبكرة للنطق واللغة والتغذية

Overview / نظرة عامة

This appendix presents the complete 25-item questionnaire used in the study to assess parental knowledge of early speech, language, and feeding red flags in children aged 0-3 years. The quiz is organized into five developmental domains and includes multiple-choice questions with explanatory feedback for each item.

يقدم هذا الملحق الاستبيان الكامل المكون من 25 عنصراً المستخدم في الدراسة لتقييم معرفة الوالدين بعلامات التحذير المبكرة للنطق واللغة والتغذية لدى الأطفال الذين تتراوح أعمارهم بين 0-3 سنوات. يتم تنظيم الاختبار في خمس مجالات نمائية ويتضمن أسئلة متعددة الخيارات مع ملاحظات توضيحية لكل عنصر.

Item-to-Domain Mapping / تعيين العناصر إلى المجالات

| Domain / المجال | Items / العناصر | Number of Items / عدد العناصر |
| --- | --- | --- |
| Speech and Language Development / تطور النطق واللغة | 1-8 | 8 |
| Feeding and Swallowing / التغذية والبلع | 9-14 | 6 |
| Hearing and Interaction / السمع والتفاعل | 15-18 | 4 |
| Social and Cognitive Communication / التواصل الاجتماعي والمعرفي | 19-24 | 6 |
| Professional Awareness / الوعي بالمهنة | 25 | 1 |
| TOTAL / الإجمالي |  | 25 |

Scoring Instructions / تعليمات التصحيح

- Correct answer / الإجابة الصحيحة: 1 point / نقطة واحدة
- Incorrect answer / الإجابة غير الصحيحة: 0 points / صفر نقطة
- Total possible score / إجمالي النقاط الممكنة: 25 points / 25 نقطة
- Scoring formula / صيغة التصحيح: (Number of correct answers / 25) × 100 = Percentage score

SECTION 1: SPEECH AND LANGUAGE DEVELOPMENT / القسم الأول: تطور النطق واللغة

(Items 1-8 / العناصر 1-8)

Item 1

Arabic Question / السؤال بالعربية: عندما يكون طفلك في عمر شهرين إلى ثلاثة أشهر ونادرًا ما يُصدر أصوات المناغاة، ماذا قد يعني ذلك؟

English Translation: When your child is 2-3 months old and rarely makes cooing sounds, what might that mean?

Options / الخيارات:

- A) بعض الأطفال يبدأون لاحقًا ولا داعي للقلق / Some children start later and there's no need to worry
- B) قد يكون الطفل كثير النوم في النهار / The child may be sleeping a lot during the day
- C) قد يحتاج الأمر إلى متابعة تطور الأصوات المبكرة ✅ / May need monitoring of early sound development ✅
- D) قد يحدّ ألم التسنين من الأصوات / Teething pain may limit sounds

Correct Answer / الإجابة الصحيحة: C

Professional Feedback / الملاحظات المهنية: المناغاة المبكرة تدل على تحكم الطفل بصوته، وغيابها قد يشير إلى حاجة للملاحظة أو الفحص.

Early cooing indicates the child's control over their voice. Its absence may indicate a need for monitoring or evaluation.

Item 2

Arabic Question / السؤال بالعربية: إذا كان طفلك في عمر سبعة أشهر ولم يبدأ بالمناغاة مثل "با با" أو "ما ما"، ما الذي قد يشير إليه ذلك؟

English Translation: If your child is 7 months old and has not started babbling like "ba ba" or "ma ma," what might that indicate?

Options / الخيارات:

- A) يحتاج الطفل إلى مزيد من التفاعل اللفظي / The child needs more verbal interaction
- B) قد يبطئ التسنين من المناغاة / Teething may slow babbling
- C) تأخر في السمع أو اللغة ✅ / Delay in hearing or language ✅
- D) الطفل خجول حول الآخرين / The child is shy around others

Correct Answer / الإجابة الصحيحة: C

Professional Feedback / الملاحظات المهنية: عدم ظهور المناغاة بعد عمر تسعة أشهر يُعد علامة مبكرة لمشكلة في السمع أو اللغة.

The absence of babbling after 9 months is an early sign of a hearing or language problem.

Item 3

Arabic Question / السؤال بالعربية: إذا أتم طفلك عامه الأول ولم يبدأ بنطق كلمات واضحة بعد، ما الإجراء الأنسب؟

English Translation: If your child has completed their first year and has not started saying clear words, what is the most appropriate action?

Options / الخيارات:

- A) الانتظار حتى يبلغ عامين / Wait until they reach two years old
- B) مراجعة أخصائي النطق واللغة ✅ / Consult a speech-language pathologist ✅
- C) تشجيع الكلام وإعادة التقييم لاحقًا / Encourage speech and reassess later
- D) سؤال آباء آخرين عن تجاربهم / Ask other parents about their experiences

Correct Answer / الإجابة الصحيحة: B

Professional Feedback / الملاحظات المهنية: من المتوقع أن يقول الطفل كلمة أو كلمتين على الأقل في عمر سنة.

It is expected that the child should say at least one or two words by age one.

Item 4

Arabic Question / السؤال بالعربية: إذا كان طفلك في عمر ثمانية عشر شهرًا ولا يقول سوى "ماما" و"بابا"، ماذا ينبغي على الوالدين فعله؟

English Translation: If your child is 18 months old and only says "mama" and "baba," what should parents do?

Options / الخيارات:

- A) الانتظار حتى تزداد المفردات طبيعيًا / Wait until vocabulary increases naturally
- B) تشجيع الطفل على الكلام في المنزل / Encourage the child to speak at home
- C) تشجيع التفاعل والتوجه لتقييم لغوي ✅ / Encourage interaction and seek language evaluation ✅
- D) التركيز على المهارات الحركية فقط / Focus only on motor skills

Correct Answer / الإجابة الصحيحة: C

Professional Feedback / الملاحظات المهنية: في عمر 18 شهرًا يجب أن يحاول الطفل قول 3 كلمات أو أكثر غير "ماما" و"بابا".

At 18 months, the child should attempt to say 3 or more words besides "mama" and "baba."

Item 5

Arabic Question / السؤال بالعربية: إذا كان الطفل في عمر سنتين لا يستخدم سوى كلمات مفردة، ماذا يجب على الوالدين فعله؟

English Translation: If the child is 2 years old and only uses single words, what should parents do?

Options / الخيارات:

- A) زيادة وقت مشاهدة الفيديوهات التعليمية / Increase time watching educational videos
- B) طلب تقييم لغوي لدى أخصائي النطق واللغة ✅ / Request a language evaluation from a speech-language pathologist ✅
- C) الانتظار حتى مرحلة الروضة / Wait until preschool
- D) سؤال الطبيب عن فيتامينات مفيدة للنطق / Ask the doctor about vitamins for speech

Correct Answer / الإجابة الصحيحة: B

Professional Feedback / الملاحظات المهنية: من الطبيعي أن يبدأ الطفل في عمر سنتين بتكوين جمل قصيرة من كلمتين.

It is normal for a child at age 2 to start forming short two-word sentences.

Item 6

Arabic Question / السؤال بالعربية: إذا كان كلام طفلك في عمر ثلاث سنوات صعب الفهم على الآخرين، ماذا يعني ذلك؟

English Translation: If your child's speech at age 3 is difficult for others to understand, what does that mean?

Options / الخيارات:

- A) أمر طبيعي لأن الغرباء لا يفهمون الأطفال بسهولة / Normal because strangers don't easily understand children
- B) قد يحتاج إلى دعم لتحسين وضوح النطق ✅ / May need support to improve speech clarity ✅
- C) سيتحسن الكلام تلقائيًا مع الوقت / Speech will improve automatically over time
- D) الطفل خجول أمام الآخرين / The child is shy in front of others

Correct Answer / الإجابة الصحيحة: B

Professional Feedback / الملاحظات المهنية: في عمر ثلاث سنوات يجب أن يكون كلام الطفل مفهومًا بنسبة 75%.

At age 3, the child's speech should be understood 75% of the time.

Item 7

Arabic Question / السؤال بالعربية: إذا لم يستجب طفلك عندما يُنادى باسمه في عمر سنة واحدة، ما الذي قد يشير إليه ذلك؟

English Translation: If your child does not respond when called by name at age 1, what might that indicate?

Options / الخيارات:

- A) احتمال وجود مشكلة في السمع أو الانتباه ✅ / Possible hearing or attention problem ✅
- B) نبرة الصوت منخفضة / The voice tone is low
- C) الطفل منشغل بالألعاب / The child is busy with toys
- D) الطفل عنيد ولا يريد التفاعل / The child is stubborn and doesn't want to interact

Correct Answer / الإجابة الصحيحة: A

Professional Feedback / الملاحظات المهنية: من المتوقع أن يستجيب الطفل لاسمه بانتظام في عمر سنة واحدة.

It is expected that the child responds to their name regularly at age one.

Item 8

Arabic Question / السؤال بالعربية: إذا طُلب من طفلك تنفيذ أمر بسيط مثل "أعطني الكرة" في عمر ثمانية عشر شهرًا ولم يستجب، ماذا قد يعني ذلك؟

English Translation: If your child is asked to follow a simple command like "give me the ball" at 18 months and doesn't respond, what might that mean?

Options / الخيارات:

- A) تأخر في فهم اللغة ✅ / Delay in language comprehension ✅
- B) ضعف في المهارات الحركية / Weakness in motor skills
- C) تشتت انتباه مؤقت / Temporary attention distraction
- D) أمر طبيعي حتى عمر سنتين / Normal until age two

Correct Answer / الإجابة الصحيحة: A

Professional Feedback / الملاحظات المهنية: من المفترض أن يبدأ الطفل في فهم الأوامر البسيطة في هذا العمر.

The child is expected to begin understanding simple commands at this age.

SECTION 2: FEEDING AND SWALLOWING / القسم الثاني: التغذية والبلع

(Items 9-14 / العناصر 9-14)

Item 9

Arabic Question / السؤال بالعربية: إذا كان طفلك يسعل أو يختنق أثناء الرضاعة أو الأكل، ماذا يجب أن تفعل؟

English Translation: If your child coughs or chokes during breastfeeding or eating, what should you do?

Options / الخيارات:

- A) جرّب حلمة بطيئة التدفق / Try a slow-flow nipple
- B) مراجعة الطبيب أو أخصائي النطق والبلع ✅ / Consult a doctor or speech-language pathologist specializing in swallowing ✅
- C) تقديم طعام أثخن / Offer thicker food
- D) الانتظار لمعرفة إن تحسّن الوضع / Wait to see if the situation improves

Correct Answer / الإجابة الصحيحة: B

Professional Feedback / الملاحظات المهنية: السعال أو الاختناق المتكرر علامة مبكرة لصعوبة البلع.

Frequent coughing or choking is an early sign of swallowing difficulty.

Item 10

Arabic Question / السؤال بالعربية: إذا كان طفلك يميل ظهره أو يتقيأ بشكل متكرر أثناء الرضاعة، ماذا يمكن أن يشير ذلك إليه؟

English Translation: If your child arches their back or vomits repeatedly during feeding, what might that indicate?

Options / الخيارات:

- A) أمر طبيعي إذا أكل بسرعة / Normal if eating quickly
- B) احتمال وجود ارتجاع أو مشكلة في البلع ✅ / Possible reflux or swallowing problem ✅
- C) رد فعل لطعم الحليب / Reaction to milk taste
- D) مغص مؤقت / Temporary colic

Correct Answer / الإجابة الصحيحة: B

Professional Feedback / الملاحظات المهنية: هذه العلامات تتطلب تقييمًا من طبيب الأطفال أو أخصائي النطق والبلع.

These signs require evaluation by a pediatrician or speech-language pathologist specializing in swallowing.

Item 11

Arabic Question / السؤال بالعربية: إذا كان الطفل في عمر سنة يرفض الأطعمة الصلبة ويقبل فقط المهروسة، هل هذا طبيعي؟

English Translation: If a 1-year-old child refuses solid foods and only accepts pureed foods, is this normal?

Options / الخيارات:

- A) نعم، يفضل بعض الأطفال الأطعمة الطرية / Yes, some children prefer soft foods
- B) لا، يحتاج إلى تقييم تغذية ✅ / No, needs nutritional evaluation ✅
- C) يفضل تقديم السوائل فقط لفترة / Prefer offering only liquids for a while
- D) قد يكون بسبب التسنين / May be due to teething

Correct Answer / الإجابة الصحيحة: B

Professional Feedback / الملاحظات المهنية: معظم الأطفال يستطيعون تناول الأطعمة ذات القوام في عمر سنة تقريبًا.

Most children can eat textured foods around age one.

Item 12

Arabic Question / السؤال بالعربية: إذا لاحظت خروج الحليب من أنف طفلك أثناء الرضاعة، ماذا قد يعني ذلك؟

English Translation: If you notice milk coming out of your child's nose during feeding, what might that mean?

Options / الخيارات:

- A) وجود مشكلة في سقف الحلق أو صعوبة في البلع ✅ / Problem with the palate or swallowing difficulty ✅
- B) شرب الطفل بسرعة / The child is drinking too fast
- C) ارتجاع بسيط غير ضار / Simple harmless reflux
- D) وضعية رضاعة غير صحيحة / Incorrect feeding position

Correct Answer / الإجابة الصحيحة: A

Professional Feedback / الملاحظات المهنية: قد تكون علامة على وجود شق في سقف الحلق أو ضعف في التنسيق أثناء البلع.

May indicate a cleft palate or weakness in coordination during swallowing.

Item 13

Arabic Question / السؤال بالعربية: إذا لم يتمكن الطفل من الشرب من الكوب بعد عمر ثمانية عشر شهرًا، ماذا يعني ذلك؟

English Translation: If the child cannot drink from a cup after 18 months, what does that mean?

Options / الخيارات:

- A) تأخر في المهارات الفموية الحركية ✅ / Delay in oral motor skills ✅
- B) تفضيل للرضاعة فقط / Preference for nursing only
- C) مرحلة تسنين متأخرة / Late teething phase
- D) طبيعي لبعض الأطفال / Normal for some children

Correct Answer / الإجابة الصحيحة: A

Professional Feedback / الملاحظات المهنية: يجب الانتقال من الرضاعة إلى الكوب في عمر 12–18 شهرًا.

Transition from nursing to cup should occur between 12-18 months.

Item 14

Arabic Question / السؤال بالعربية: إذا كانت وجبات طفلك تستغرق أكثر من ثلاثين دقيقة ويصاحبها سعال أو تقيؤ، ماذا يجب أن تفعل الأسرة؟

English Translation: If your child's meals take more than 30 minutes and are accompanied by coughing or vomiting, what should the family do?

Options / الخيارات:

- A) تقديم أطعمة أكثر ليونة / Offer softer foods
- B) مراجعة أخصائي لتقييم البلع أو التغذية ✅ / Consult a specialist for swallowing or nutrition evaluation ✅
- C) السماح بوقت أطول للوجبات / Allow more time for meals
- D) تغيير الكرسي المستخدم أثناء الأكل / Change the chair used during eating

Correct Answer / الإجابة الصحيحة: B

Professional Feedback / الملاحظات المهنية: الوجبات الطويلة أو المجهدة تعتبر من مؤشرات صعوبات البلع أو التغذية.

Long or stressful meals are indicators of swallowing or feeding difficulties.

SECTION 3: HEARING AND INTERACTION / القسم الثالث: السمع والتفاعل

(Items 15-18 / العناصر 15-18)

Item 15

Arabic Question / السؤال بالعربية: إذا لم يتفاعل طفلك في عمر ثلاثة أشهر مع الأصوات العالية، ماذا قد يعني ذلك؟

English Translation: If your child does not react to loud sounds at 3 months, what might that mean?

Options / الخيارات:

- A) الطفل كان متعبًا / The child was tired
- B) احتمال وجود ضعف في السمع ✅ / Possible hearing loss ✅
- C) ردة الفعل تظهر لاحقًا / The reaction appears later
- D) الغرفة كانت صاخبة جدًا / The room was too noisy

Correct Answer / الإجابة الصحيحة: B

Professional Feedback / الملاحظات المهنية: ينتبه الطفل الطبيعي للأصوات العالية منذ الأشهر الأولى.

A typically developing child responds to loud sounds from the early months.

Item 16

Arabic Question / السؤال بالعربية: إذا كان طفلك في عمر ستة أشهر ولا يلتفت نحو صوتك، ماذا ينبغي أن تفعل؟

English Translation: If your child is 6 months old and does not turn toward your voice, what should you do?

Options / الخيارات:

- A) تحديد موعد لفحص السمع ✅ / Schedule a hearing test ✅
- B) التحدث بصوت أعلى / Speak louder
- C) سؤال الأقارب إن كان ذلك وراثيًا / Ask relatives if it's hereditary
- D) الانتظار حتى عمر سنة / Wait until age one

Correct Answer / الإجابة الصحيحة: A

Professional Feedback / الملاحظات المهنية: الالتفات نحو الصوت من المهارات الأساسية التي تظهر بحلول عمر ستة أشهر.

Turning toward sound is a fundamental skill that appears by 6 months.

Item 17

Arabic Question / السؤال بالعربية: إذا كان الطفل في عمر سنتين يفضل صوت التلفاز العالي جدًا، ماذا يمكن أن يعني ذلك؟

English Translation: If a 2-year-old child prefers very loud TV volume, what might that mean?

Options / الخيارات:

- A) ضعف في السمع ✅ / Hearing loss ✅
- B) تقليد للبالغين / Imitating adults
- C) حب للأصوات المرتفعة / Love of loud sounds
- D) ذوق خاص في الموسيقى / Special taste in music

Correct Answer / الإجابة الصحيحة: A

Professional Feedback / الملاحظات المهنية: الفحص السمعي المبكر مهم جدًا.

Early hearing screening is very important.

Item 18

Arabic Question / السؤال بالعربية: كيف يمكن أن تؤثر التهابات الأذن المتكررة على تطور الطفل؟

English Translation: How can repeated ear infections affect a child's development?

Options / الخيارات:

- A) على الشهية / On appetite
- B) على النوم / On sleep
- C) على تطور النطق واللغة ✅ / On speech and language development ✅
- D) على النمو الجسدي / On physical growth

Correct Answer / الإجابة الصحيحة: C

Professional Feedback / الملاحظات المهنية: تراكم السوائل في الأذن الوسطى يؤثر مؤقتًا على السمع ويؤخر تطور الكلام.

Fluid accumulation in the middle ear temporarily affects hearing and delays speech development.

SECTION 4: SOCIAL AND COGNITIVE COMMUNICATION / القسم الرابع: التواصل الاجتماعي والمعرفي

(Items 19-24 / العناصر 19-24)

Item 19

Arabic Question / السؤال بالعربية: إذا كان طفلك في عمر ثلاثة أشهر لا يبتسم عند التحدث إليه أو عند رؤيتك، ماذا قد يعني ذلك؟

English Translation: If your child is 3 months old and does not smile when spoken to or when seeing you, what might that mean?

Options / الخيارات:

- A) متعب / Tired
- B) احتمال وجود تأخر في التطور ✅ / Possible developmental delay ✅
- C) يفضل الهدوء / Prefers quiet
- D) يتعرض لمحفزات كثيرة / Exposed to too many stimuli

Correct Answer / الإجابة الصحيحة: B

Professional Feedback / الملاحظات المهنية: يجب أن يظهر "الابتسام الاجتماعي" في عمر شهرين تقريبًا.

"Social smiling" should appear around 2 months of age.

Item 20

Arabic Question / السؤال بالعربية: إذا كان الطفل في عمر سنة لا ينظر إلى وجهك أو لا يشارك في ألعاب التفاعل مثل "إخفاء الوجه"، ماذا قد يشير ذلك إليه؟

English Translation: If a 1-year-old child does not look at your face or does not participate in interactive games like "peek-a-boo," what might that indicate?

Options / الخيارات:

- A) الطفل خجول / The child is shy
- B) تأخر في التواصل الاجتماعي ✅ / Delay in social communication ✅
- C) لا يحب الألعاب / Doesn't like games
- D) يشعر بالنعاس / Feels sleepy

Correct Answer / الإجابة الصحيحة: B

Professional Feedback / الملاحظات المهنية: غياب التفاعل الاجتماعي قد يكون مؤشرًا مبكرًا للتوحد.

Lack of social interaction may be an early indicator of autism.

Item 21

Arabic Question / السؤال بالعربية: إذا لم يبدأ الطفل في عمر سنتين باللعب التخيلي مثل إطعام الدمية، ماذا يعني ذلك؟

English Translation: If a 2-year-old child has not started pretend play like feeding a doll, what does that mean?

Options / الخيارات:

- A) تأخر في التطور الاجتماعي أو المعرفي ✅ / Delay in social or cognitive development ✅
- B) يفضل اللعب الحركي / Prefers physical play
- C) لا يملك ألعابًا كافية / Doesn't have enough toys
- D) يشعر بالتعب أثناء اللعب / Feels tired during play

Correct Answer / الإجابة الصحيحة: A

Professional Feedback / الملاحظات المهنية: اللعب التخيلي دليل على النمو اللغوي والتفكير الرمزي.

Pretend play is evidence of language growth and symbolic thinking.

Item 22

Arabic Question / السؤال بالعربية: إذا كان الطفل لا يقلد الأصوات أو الأفعال في عمر سنتين، ما الذي قد يشير إليه ذلك؟

English Translation: If a 2-year-old child does not imitate sounds or actions, what might that indicate?

Options / الخيارات:

- A) خطر وجود تأخر لغوي ✅ / Risk of language delay ✅
- B) استقلالية قوية / Strong independence
- C) قلة الاهتمام بالآخرين / Lack of interest in others
- D) أمر طبيعي في هذا العمر / Normal at this age

Correct Answer / الإجابة الصحيحة: A

Professional Feedback / الملاحظات المهنية: التقليد من أهم المهارات لتعلم اللغة.

Imitation is one of the most important skills for language learning.

Item 23

Arabic Question / السؤال بالعربية: إذا كان الطفل قد فقد كلمات أو مهارات تعلمها سابقًا، ماذا يعني ذلك؟

English Translation: If a child has lost words or skills they previously learned, what does that mean?

Options / الخيارات:

- A) علامة إنذار لتراجع نمائي ✅ / Warning sign of developmental regression ✅
- B) ملل من الكلام / Bored with speaking
- C) خوف من الغرباء / Fear of strangers
- D) اختلاف طبيعي / Natural variation

Correct Answer / الإجابة الصحيحة: A

Professional Feedback / الملاحظات المهنية: فقدان المهارات المكتسبة يستدعي مراجعة طبية عاجلة.

Loss of acquired skills requires urgent medical consultation.

Item 24

Arabic Question / السؤال بالعربية: إذا كان الطفل في عمر ثلاث سنوات نادرًا ما يتحدث أو لا يشارك في محادثات بسيطة، ماذا يشير ذلك إليه؟

English Translation: If a 3-year-old child rarely speaks or does not participate in simple conversations, what does that indicate?

Options / الخيارات:

- A) تأخر لغوي أو صعوبة في التواصل الاجتماعي ✅ / Language delay or social communication difficulty ✅
- B) يفضل اللعب منفردًا / Prefers playing alone
- C) خجول فقط / Just shy
- D) لا يهتم بالكلام / Not interested in speaking

Correct Answer / الإجابة الصحيحة: A

Professional Feedback / الملاحظات المهنية: في عمر ثلاث سنوات يجب أن يستمتع الطفل بالمحادثات البسيطة.

At age 3, a child should enjoy simple conversations.

SECTION 5: PROFESSIONAL AWARENESS / القسم الخامس: الوعي بالمهنة

(Item 25 / العنصر 25)

Item 25

Arabic Question / السؤال بالعربية: عند ملاحظتك لتأخر في النطق أو الأكل لدى طفلك، من المختص الذي يجب مراجعته؟

English Translation: When you notice a delay in speech or eating in your child, which specialist should you consult?

Options / الخيارات:

- A) طبيب الأطفال للنصيحة العامة / Pediatrician for general advice
- B) أخصائي النطق واللغة ✅ / Speech-language pathologist ✅
- C) الأخصائي النفسي للسلوك / Psychologist for behavior
- D) معلم المدرسة للتحضير الدراسي / Teacher for academic preparation

Correct Answer / الإجابة الصحيحة: B

Professional Feedback / الملاحظات المهنية: أخصائيو النطق واللغة هم المختصون في تقييم وعلاج اضطرابات التواصل والبلع بالتعاون مع أطباء الأطفال.

Speech-language pathologists are specialists in evaluating and treating communication and swallowing disorders in collaboration with pediatricians.

Scoring Key / مفتاح التصحيح

| Item / العنصر | Correct Answer / الإجابة الصحيحة | Domain / المجال |
| --- | --- | --- |
| 1 | C | Speech and Language Development |
| 2 | C | Speech and Language Development |
| 3 | B | Speech and Language Development |
| 4 | C | Speech and Language Development |
| 5 | B | Speech and Language Development |
| 6 | B | Speech and Language Development |
| 7 | A | Speech and Language Development |
| 8 | A | Speech and Language Development |
| 9 | B | Feeding and Swallowing |
| 10 | B | Feeding and Swallowing |
| 11 | B | Feeding and Swallowing |
| 12 | A | Feeding and Swallowing |
| 13 | A | Feeding and Swallowing |
| 14 | B | Feeding and Swallowing |
| 15 | B | Hearing and Interaction |
| 16 | A | Hearing and Interaction |
| 17 | A | Hearing and Interaction |
| 18 | C | Hearing and Interaction |
| 19 | B | Social and Cognitive Communication |
| 20 | B | Social and Cognitive Communication |
| 21 | A | Social and Cognitive Communication |
| 22 | A | Social and Cognitive Communication |
| 23 | A | Social and Cognitive Communication |
| 24 | A | Social and Cognitive Communication |
| 25 | B | Professional Awareness |

Notes for Administration / ملاحظات الإدارة

1. Language / اللغة: This quiz is designed for Arabic-speaking parents and caregivers. It should be administered in Arabic to ensure comprehension.
2. Time / الوقت: The quiz typically takes 10-12 minutes to complete.
3. Format / الصيغة: The quiz can be administered in paper-and-pencil format or digitally through an online platform or gamified application.
4. Feedback / التعليقات: Participants should receive immediate feedback after each question, including the correct answer and explanatory information to support learning.
5. Use / الاستخدام: This quiz is designed for educational purposes to increase parental awareness of early developmental red flags and promote early identification of developmental concerns.
6. Scoring / التصحيح: Scores should be calculated as a percentage (number of correct answers / 25 × 100). Average scores can be compared between pre-test and post-test administrations to assess learning gains.
7. Instructions / التعليمات:
   - يرجى قراءة كل سؤال بعناية واختيار إجابة واحدة فقط.
   - يقدم كل سؤال موقفًا واقعيًا يواجهه الوالدان في الحياة اليومية.
   - بعد الإجابة، ستظهر تغذية راجعة فورية تشرح الإجابة الصحيحة.
   - الإجابات مجهولة الهوية وتُستخدم لأغراض تعليمية وبحثية فقط.
   - لا توجد نتائج صحيحة أو خاطئة- الهدف هو التعلم واكتساب الوعي.
